# McKillip Veterinary College

**Published:** 1897-05

**Authors:** 


### McKILLIP VETERINARY COLLEGE.

The commencement exercises of the college were held in Assembly Hall, Tuesday evening, March 30th. After prayer by Rev. Dr.

Staff, Acting Dean Schoenleber addressed the students, followed by a salutatory by Dr. E. Merillat. An address was also made by Dr. E. M. Reading; "Class Prophecy," by S. Hutson Caldwell; "Class Poem," by W. S. Powell; "Class History," by O. R. Dubia. After the valedictory by E. H. Brown the degree of M.D.V. was conferred by President McKillip on the following graduates: Ernest H. Brown, Hawarden, Iowa; John B. Boomer, Chicago, Ill.; John H. Crawford, Racine, Wis.; S. Hutson Caldwell, Chicago, Ill.; Charles N. Ferrier, Jamestown, N. D.; E. Merillat, Toledo, Ohio; W. S. Powell, Sparta, Wis.; O. R. Dubia, Chicago, Ill.; George J. Tyner, Paradise, Ind.; and Harry O. Davis, Chicago, Ill.

The exercises were witnessed by a large number of invited guests.

